# Industry Competition, Corporate Governance, and Voluntary Disclosure of Greenhouse Gas Emissions Information: Evidence from South Korea

**DOI:** 10.3390/ijerph192316272

**Published:** 2022-12-05

**Authors:** Hyunah Lee, Jaehong Lee

**Affiliations:** 1 School of Business, Gachon University, Seongnam 13120, Republic of Korea; 2 Major in Accounting/Taxation, Division of Business Administration, Kyonggi University, Suwon 16227, Republic of Korea

**Keywords:** industry competition, corporate governance, greenhouse gas emissions information

## Abstract

This study investigates the relationship between industry competition and managers’ voluntary disclosure policies and examined how the corporate governance structure affects this relationship in South Korean companies. The fiercer the competition within the industry to which the company belongs, the higher the incentive for managers to perform strategic actions to improve their competition status. This increase in the strategic incentives of managers can be seen through voluntary disclosure policies. The empirical results of this study are as follows. First, it was found that there was a negative relationship between the degree of industry competition and the level of voluntary disclosure of greenhouse gas emissions information. This means that managers perform less disclosure to maximize the value of the company because the more competition within the industry intensifies, the higher the proprietary cost of disclosing information on greenhouse gas emissions information. Second, it was found that the corporate governance structure weakened the relationship between the degree of industry competition and the level of corporate voluntary disclosure. These results can be interpreted as that a good governance structure supports such managers’ disclosure decisions because managers are more likely to choose disclosure policies to maximize the value of the company than personal benefits even in the fierce industry competition.

## 1. Introduction 

This study aims to understand the relationship between industry-level competition as a corporate environmental factor and voluntary disclosure policies on greenhouse gas emissions, and to examine how corporate governance affects these relationships. Competition serves as a discipline to encourage managers to avoid bankruptcy and to attempt to maintain their position, as increasing industry competition can exacerbate corporate profitability, which may lead to CEO turnover [1,2,3]. In addition, in the presence of a competitor, the performance of the other corporate managers is compared and evaluated based on the interests of the competitor, which can reduce managerial negligence and ultimately induce the corporate profits to increase voluntarily. This role of competition leads to a consensus in interests between managers and shareholders in highly competitive industries by reducing agency problems [1,4,5].

As a result, managers who face fierce competition are likely to establish management strategies in the direction of maximizing the value of the company rather than their own personal interests. In addition, since increasing competition is likely to worsen corporate profitability and increases the risk of corporate bankruptcy, the fiercer competition in the industry gives managers a high incentive to take strategic actions to increase profits and lower costs to maintain a competitive advantage among competitors. This increase in the strategic incentives of managers can be reflected in the company’s managerial strategy in various forms. The corporate disclosure policy on greenhouse gas emissions could be especially sensitive to the managers as it can affect the corporate competitive position if the competitor responds strategically to the disclosed information.

Traditionally, disclosures have been reported to provide economic benefits to the firm by increasing liquidity in stocks and reducing costs of capital by reducing uncertainties caused by information asymmetry among outside investors in the capital market [6,7,8,9,10]. However, the disclosure of corporate private information provides competitors with the opportunity to take strategic action with the disclosed information, which also threatens the competitive position of the disclosure entity [11]. These disclosure-related costs are referred to as proprietary costs, and previous studies have reported that managers choose disclosure levels at their discretion, taking into account the benefits and costs of disclosure, depending on competitive strength [11,12,13,14]. Despite the fact that considering the competitive environment of the industry to which the company belongs is important, few studies have examined the relationship between the degree of competition and the level of voluntary disclosure. Additionally, some previous studies reported inconsistent and mixed empirical results on the relationship between industry competition and comprehensive disclosure [15,16,17]. Therefore, this study aims to empirically verify the relationship between industry-level competition and the level of voluntary disclosure of greenhouse gas emissions information.

Next, this study examines how corporate governance affects the relationship between industry-level competition and the voluntary disclosure of greenhouse gas emissions information. Previous studies have reported that there is a complementary relationship between competition and corporate governance and that the interaction between them leads to the efficient monitoring of managers, lowering agency problems, and further maximizing corporate value [18]. This means that the role of competition that governs managers to do their best to maximize corporate value and the function of corporate governance as an institutional device in which stakeholders monitor and control managers to maximize corporate value are combined, which eventually leads managers to act efficiently. In this way, the interaction between competition and corporate governance may induce the optimal decision-making of managers to maximize the value of the company, which can also affect the manager’s voluntary disclosure policy. However, as there are no studies examining how the interaction between industry-level competition and corporate governance affects the corporate voluntary disclosure of greenhouse gas emissions information, this study intends to conduct an empirical analysis of this relationship.

This paper is timely since it potentially provides useful policy implications. The interest in climate change and environmental protection is growing rapidly [19,20,21,22,23]. According to the ‘Global Risk Report 2021’ published by the World Economic Forum (WEF), extreme weather, climate action failures, human environmental damage, infectious diseases, and biodiversity loss are mentioned as serious risk factors that mankind can face [22]. In light of these global trends, the Korean government is implementing policies to transform the economy into a low-energy and low-carbon economy through the development of greenhouse gas-decreasing technologies and the introduction of green infrastructure investment [22]. However, domestic experts assess that Korean companies that do not require mandatory disclosure of carbon emissions are passive in responding to the climate crisis. Specifically, the Financial Services Commission will require KOSPI-listed companies to disclose ESG information sequentially; the “G” report containing governance information will be mandatory from 2026, and the “S” report containing social responsibility information and the “E” report, which is environmental information, will be mandatory from 2030. In other words, the Financial Services Commission plans to leave the disclosure to its autonomy before the mandatory period. Therefore, companies that disclose various information such as carbon emissions are only large companies with many overseas investors, and most companies with less “pressure” from overseas investors only disclose information they want to disclose. In other words, if a company exploits it, it is possible to make it look like an eco-friendly company by disclosing only favorable information. In this situation, this study will be an interesting study to examine the role of industry competition and corporate governance in selecting whether companies voluntarily disclose carbon emissions information.

This study consists of the following order. The introduction raises questions and presents contributions from previous studies. Section 2 examines the theoretical background and establishes the hypothesis of the study. Section 3 presents the measurement method and definition of the variables used in the model, followed by the sample selection procedure. Section 4 presents the results of empirical analysis, and Section 5 summarizes the conclusions and implications of this study.

## 2. Backgrounds and Hypotheses

In the past, competition-related studies have mainly examined the effect of market competition on the price or resource allocation efficiency of products by economists, but recently, studies have been conducted to analyze the relationship between competition and managerial decision-making. These studies have argued that companies in highly competitive industries have a high risk of bankruptcy due to deteriorating profitability and a high risk of managerial replacement, leading managers to do their best to protect their positions [1,2,3]. It also reported that if a competitor exists in the same industry, it is easier for outside investors to monitor managerial behavior in competitive industries than in monopolistic industries because it can evaluate the performance of the competitor based on their profits [3,4]. As competition acts as a mechanism for regulating and monitoring managers to do their best to maximize corporate value, the more intense competition results in an alignment of interests between managers and stakeholders, thereby reducing agency problems [1,3,5,24].

Faced with such fierce competition, the manager of a company will establish a management strategy in the direction of maximizing corporate value rather than performing opportunistic actions for his or her personal interests. In addition, since the increase in competition worsens the company’s profitability and increases the risk of bankruptcy, the fiercer the competition in the industry, the stronger the incentive for the manager to make strategic choices to secure profitability. In particular, since disclosure is a sensitive issue to managers as it can directly affect the competitive position of the company, the manager’s voluntary disclosure policy is expected to vary depending on the competition situation faced by the company.

Previous studies related to disclosure reported that disclosure provides economic benefits to external investors by reducing uncertainty due to information asymmetry in the capital market [6,7,8] and reducing capital costs [9,10,25,26]. However, disclosure can also have costs because it allows competitors to take strategic action using disclosed information, which can result in threats to the competitive status of the disclosure entity [11]. Therefore, managers will strategically establish disclosure policies in consideration of the competition situation faced by the company. In other words, if it is determined that the benefits are greater by comparing the benefits and costs of disclosure, it will actively disclose information about the entity; otherwise, it will try to hide the information. As previous studies have confirmed that the size of the proprietary cost varies depending on the degree of competition [11,13,14,27,28], eventually, it can be expected that the manager’s voluntary disclosure policy will be influenced by the level of competition in the industry.

In summary, the fiercer the competition in the industry, the more incentive for managers who feel threatened by worsening profitability and the high risk of bankruptcy to carry out management strategies that lower costs and maximize corporate value. In particular, regarding the manager’s choice of disclosure policy, disclosure has both benefits and costs. If competition in the industry is fierce, it is more likely to be threatened by exposing internal information to other competitors. Accordingly, managers will avoid providing corporate information by judging that the stronger the competition is, the higher the cost than the benefit of disclosure. Therefore, it can be expected that the more competition in the industry intensifies, the less voluntary disclosure a manager will provide.

On the other hand, the level of voluntary disclosure of companies in highly competitive industries may be higher than those belonging to industries that are not. In general, in industries with low competition, companies report high profitability, and managers want to maintain excess return and high market share. The disclosure of companies in less competitive industries can be avoided by managers because it can harm these excess gains and market returns [12,29]. In addition, if competition intensifies, stakeholders including shareholders will require the firm to carry out a higher level of disclosure policy, as the risk to investment increases due to worsening profitability. In this case, competition in the industry and the level of voluntary disclosure of companies are expected to have a positive relationship.

As discussed above, there is a possibility that the increase in competition will increase or decrease the incentive for voluntary disclosure by managers. Therefore, since the relationship between the degree of competition in the industry and the voluntary disclosure level of the company is expected to be possible in both directions, this study establishes a hypothesis in the form of a null hypothesis as follows.

**Hypothesis** **1.** *The level of competition within the industry will not be related to the level of voluntary disclosure of greenhouse gas emissions information.*

Next, this study examines how corporate governance affects the relationship between the degree of competition in the industry and the manager’s voluntary disclosure policy. Corporate governance refers to an institutional device that prevents agency problems caused by managers’ actions against shareholders’ interests and monitors and controls managers to maximize corporate value. Previous studies have reported that intra-industry competition works in the direction of efficiently monitoring managers through interactions with internal corporate governance to lower agency problems and further maximize corporate value [18,30,31]. This means that the role of market competition that regulates managers to do their best to maximize corporate value and the function of internal corporate governance as an institutional device in which shareholders and stakeholders monitor and control managers to maximize corporate value are combined. In this way, competition in the industry and interaction with corporate governance induce managers to make optimal decisions to maximize corporate value, so it is expected to cause differences in managers’ disclosure decision-making according to the degree of competition. Therefore, it is necessary not only to examine the relationship between industry competition and the level of voluntary disclosure of managers but also to investigate the internal corporate governance structure in their relationship.

Prior studies examining the relationship between corporate governance and voluntary disclosure suggested that good corporate governance can have a positive effect on corporate disclosure policies because it provides an institutional mechanism to reduce managerial opportunistic behavior and information asymmetry [7,32,33]. Empirical studies have also reported that companies with good governance can effectively monitor and supervise managers to prevent managers from pursuing private interests and incentives to hide them [33,34,35,36]. Thus, managers of companies with good corporate governance have incentives to expand voluntary disclosure policies on greenhouse gas emissions for the following reasons. Although the disclosure of greenhouse gas emissions information is an option, not an obligation, this information, which is voluntarily disclosed by the firm, is evaluated as reliable in the capital market [37]. Because companies voluntarily report greenhouse gas information to Carbon Disclosure Project (CDP) and the agency evaluates and manages reporting companies with common worldwide objective standards, capital market stakeholders can objectively compare greenhouse gas emissions information from similar companies in the same industry [37]. The number of companies reporting greenhouse gas emission information to the CDP worldwide is on the rise, and for this reason, the related opportunity cost that companies are responsible for if incorrect greenhouse gas emission information is disclosed will increase. According to the above studies, the better the corporate governance structure, the more information the company will disclose as stakeholders demand a higher level of disclosure to monitor the manager, regardless of the manager’s strategic disclosure choice considering the competitive situation in the company. In other words, the more companies with good governance structures, the weaker the relationship between industry competition and managers’ strategic disclosure choices.

However, the above expectation assumes that it is desirable to disclose all information about the entity to maximize its value by emphasizing only the benefits of the disclosure without taking into account the costs incurred by the disclosure. In other words, despite the existence of proprietary costs incurred according to the degree of competition in the industry to which the company belongs, the fact that a good governance structure operates to increase disclosure unconditionally simplifies the competitive environment in which the company exists. As discussed in Hypothesis 1, the disclosure of corporate information may incur more costs than benefits depending on the degree of competition, so inducing disclosure by managers without such consideration may hinder the value of the company, in particular, because the more the competitive agency problems of a manager decreases, the more likely the manager’s disclosure policies are to be strategic choices to maximize corporate value over opportunistic actions to pursue private interests [1,4]. As mentioned above, the purpose of corporate governance is to ultimately maximize the value of the company, so if the governance structure is working properly, it will help managers to make optimal decisions. Therefore, it can be expected that the more companies with good corporate governance, the stronger the relationship between the degree of industry competition and the strategic disclosure choice of managers.

On the one hand, intense intra-industry competition requires managers with the ability and expertise to perform complex management activities [38] and specialized knowledge in rapid decision-making and decision-making [39]. Accordingly, companies in highly competitive industries give managers a high level of authority and discretion in making decisions so that they can respond quickly to competitive situations [38,39]. If discretion is not given to managerial decisions, the corporate market share and profitability may decline, face bankruptcy, and potentially be subject to mergers and acquisitions. Under these competitive circumstances, it would be desirable for corporate governance to play a minimal role in ensuring that managers make optimal decisions, rather than acting as a system to constrain and monitor managerial behavior [24]. Even in this case, good corporate governance can be expected to strengthen the relationship between industry competition and managers’ strategic disclosure choices.

As discussed above, the effect of the interaction between competition and corporate governance on the level of voluntary disclosure is expected to be possible in both directions. In addition, since there are no studies that have presented clear empirical analysis results for this relationship, this study establishes a hypothesis in the form of a null hypothesis as follows.

**Hypothesis** **2.** *The relationship between the degree of industry competition and the level of voluntary disclosure of greenhouse gas emissions information will not differ depending on the corporate governance structure.*

## 3. Research Design and Sample Description

### 3.1. Measurement of the Disclosure Level 

#### 3.1.1. Measurement of Voluntary Disclosure on Greenhouse Gas Emissions (VDGHG)

In this study, in order to measure managers’ voluntary disclosure decisions, whether to voluntarily disclose greenhouse gas emission information was used as a dependent variable. The Carbon Disclosure Project (CDP) was established in December 2000 with the support of 35 European financial institutions. Since then, the number of member institutions subscribed to the CDP has gradually increased, and as a result, about 5500 companies around the world are offering climate change information to investors through this institution. Currently, the CDP requires and collects accurate information on how related companies respond to major greenhouse gas emissions-related problems known as major causes of climate change on behalf of financial institutions around the world. In addition, the CDP provides information that can identify the level of climate change risk of certain companies belonging to high-risk groups in advance so that investors do not fall into danger. Specifically, if selected as a company subject to the greenhouse gas emission survey, it is possible to write answers voluntarily when the CDP survey comes every year, and if the answer is written and sent to the CDP, the contents are assessed by the method jointly developed by the CDP and PwC. In the case of Korean companies, the CDP Korean Committee has partially revised the assessment method according to the domestic situation, and the results of this evaluation will be released online through the CDP official website (http://cdproject.net (accessed on 1 April 2021) and the CDP final report. This study manually extracted and used data on greenhouse gas emission information of companies disclosed through the CDP official website.

#### 3.1.2. Measurement of Industry Competition (HHI)

Generally, the degree of competition is affected by the number of companies currently competing and relative to each other, threats to new enterprises, the bargaining power of suppliers and buyers, and the threat of substitutes [40]. Among these, industry competition, that is, the market structure, is easier to quantify than other variables, so the degree of competition is quantified based on market variables [31]. The fact that the market structure is widely used as a measurement variable for competition is not simply because it is easy to quantify. The importance of the market structure has also been emphasized in terms of the efficiency of resource allocation. For example, if any market concentration is low, this means that several companies share the market evenly, and unless there are special restrictions, existing companies are in fierce competition. In these competitive markets, the efficient allocation of resources can be expected. On the contrary, if it is an exclusive market due to the high concentration of the market, there is a high possibility that the inefficient allocation of resources will occur due to the existence of monopoly profits.

In this respect, the market structure can be used as a variable to measure the degree of competition in the market, and in fact, the market structure is used as an important criterion in implementing competition policies in various countries. In Korea, Article 3-2 of the Fair Trade Act prohibits the abuse of the market dominance of business operators, and Article 4 of the same Act estimates that a company’s market share exceeds a certain part of the market dominance.

Based on the relationship between this market structure and the degree of competition, previous studies at home and abroad use industrial concentration to measure the degree of competition [2,5,13,14]. In this study, industrial concentration was used in consideration of ease of measurement, consistency with prior studies and domestic policies, and the Herfindahl–Hirschman Index (*HHI*) was widely used in previous studies. Here, the *HHI* is a measure of industrial concentration, and assuming that industrial concentration and intra-industry competition have an inverse correlation, it can be interpreted that if the industrial concentration (*HHI*) is high in a specific industry, the degree of competition is low. In this study, *HHI* was calculated based on the Korean Standard Industrial Classification (KSIC 9) subclassification. The method of obtaining the *HHI* is as follows.

(1) $$HHI_{Jt}=\sum_{i=1}^{N}S_{ijt}^{2}$$

where, *S_ijt_* = market share of firm *i* at year *t*, which is included in *j* industry.

First of all, *HHI* has a square value of the market share of all companies operating in the same industry. An individual company’s market share is calculated by dividing the total sales by each company’s industrial sales. Next, I will square the market share of the individual entity calculated earlier and then sum up all the values. In this study, the *HHI* values are given by industry years [4,5,30]. Here, the lower the value of the *HHI*, the more market is shared by a number of competitors, while the larger the value of the *HHI*, the more concentrated the market is by a small number of large companies. In this study, in verifying the relationship between the degree of industry competition and the disclosure level of the company, the verification is performed by multiplying the *HHI* by a negative value.

#### 3.1.3. Corporate Governance Index (CG)

To measure the degree of the corporate governance index, I employ ESG score data collected from KCGS. KCGS is a semi-governmental organization that evaluates various parts of sustainability management within Korean companies for policy-making purposes. Since 2011, KCGS has been carrying out annual ESG assessments and openly disclosing ESG scores. The E score (environmental responsibility score) evaluation quantifies the planning, execution, performance management, and reporting of environmental responsibilities within firms. The S score (social responsibility score) evaluation quantifies the social responsibility of firms towards employees, customers, partners, competitors, and local communities. The G score (governance score) evaluation includes investor protection, board and auditor effectiveness, and disclosure. Among these scores, this study uses the sum of the G scores for the corporate governance variable. 

#### 3.1.4. Measurement of Earnings Quality (DA)

The equation to measure earnings quality (DA) as a control variable is as follows [41].

(2) $$\frac{TA_{t}}{A_{t1}}=\alpha_{0}+\beta_{1}\frac{1}{A_{t1}}+\beta_{2}\frac{\Delta S_{t}\;\Delta AR_{t}}{A_{t1}}+\beta_{3}\frac{PPE_{t}}{A_{t1}}+\beta_{3}ROA_{t}+\varepsilon_{t}$$

where *TA* = Net income cash flow from operations; *S* = Sales revenue; *AR* = Accounts receivables; *PPE* = Plant, property, and equipment; *ROA* = Net income/total assets; *A* = Total assets.

A cross-sectional model of discretionary accruals was employed, and it is assessed based on all industries classified by a two-digit industry code. The sample includes only companies with more than 20 firm-year observations to ensure efficient data required for estimation. The residuals calculated from the above estimation model of Equation (2) are used to measure the discretionary accruals (DA). I employed it after multiplying DA by the negative one for ease of interpretation. 

### 3.2. Research Model 

This study used the following regression model to investigate whether industry competition affects the voluntary disclosure of greenhouse gas emission information.

(3) $$\begin{array}{l} VDGHG_{t+1}=\alpha_{0}+\beta_{1}HHI_{t}+\beta_{2}SIZE_{t}+\beta_{3}LEV_{t}+\beta_{4}MTB_{t}+\beta_{5}ROA_{t}+\beta_{6}DA_{t}+\beta_{7}MO_{1}\\ +\beta_{8}FO_{t}+\beta_{9}MKT_{1}+\sum IND+\sum YR+\varepsilon_{t} \end{array}$$

where *VDGHG* = 1 if a firm voluntarily discloses greenhouse gas emissions information; 0 otherwise; *HHI* = 1 if a firm is included in a competitive industry; 0 otherwise; *SIZE* = the natural logarithm of total assets; *LEV* = total debt/total asset; *MTB* = market value of equity/book value of equity; *ROA* = net income/total asset; *DA* = earnings quality measured by discretionary accruals; *MO* = majority shareholders’ ownership; *FO* = foreign investors’ ownership; *MKT* = 1 if a firm is included in the KOSPI market; 0 otherwise.

The research model in Equation (3) incorporates proper control variables that can impact the voluntary disclosure of greenhouse gas emission information. These are firm size (*SIZE*), leverage (*LEV*), market-to-book ratio (*MTB*), return on assets (*ROA*), earnings quality (*DA*) measured by Kothari et al., majority shareholders’ ownership (*MO*), foreign ownership (*FO*), and stock market (*MKT*). The research model also includes year fixed dummies and industry-fixed effect dummies to permit for variations across firms in the matching industry-year observations. As far as industry competition affects the voluntary disclosure of carbon emission information, the coefficient of the HHI will display a significantly positive/negative number. 

Second, this study takes on the following model to examine Hypothesis 2. The key independent variable in the regression model (4) is the interaction term between the HHI and the CG. The remaining variables are the same as those defined in Equation (3), and industry and year fixed effects are also controlled in the model.

(4) $$\begin{array}{l} VDGHG_{t+1}=\alpha_{0}+\beta_{1}HHI_{t}+\beta_{2}CG_{t}+\beta_{3}HHI\times CG_{t}+\beta_{4}SIZE_{t}+\beta_{5}LEV_{t}+\beta_{6}MTB_{t}+\beta_{7}ROA_{1}\\ +\beta_{8}DA_{t}+\beta_{9}MO_{1}+\beta_{10}FO_{1}+\beta_{11}MKT_{1}+\sum IND+\sum YR+\varepsilon_{t} \end{array}$$

where, *CG* = corporate governance index by KCGS.

### 3.3. Data Selection 

Table 1 shows the collection process of data to be used for hypothesis verification. The research data used by this study include companies that settled at the end of December listed on the Korea Stock Exchange from 2014 to 2020. Specifically, the sample of this paper consists of (1) manufacturing firms excluding financial institutions, (2) companies that can receive financial statements from the FnGuide database, and (3) companies that have information on greenhouse gas emissions obtained from the CDP. Companies with incomplete data were removed, and since outliers could affect the empirical analysis results of the paper, the top and bottom 1% of each variable were winsorized. The final data selected in this way are a sample of 9406 firm-years.

## 4. Empirical Results

### 4.1. Descriptive Statistics 

Table 2 shows the descriptive statistics on the main variables that are used in this study. The average value of the voluntary disclosure of greenhouse gas emission information (VDGHG) is 0.0488, and its median is 0.0000. The mean value of the HHI is 0.5538, and its median is 1.0000. Lastly, the average value of the CG is 3.3288, which is lower than its median value of 3.3672.

Table 3 shows the Pearson correlation of the main variables. As a result, the voluntary disclosure of greenhouse gas emission information is positively related to industry competition (HHI). Furthermore, a positive relationship is presented on CG with 1% significance. Despite the significant association between the main variables, this Pearson correlation analysis is a univariate test with a limitation that does not include the other effects of control variables.

#### 4.1.1. Regression Results—Hypothesis 1

Table 4 presents the regression results on the association between industry competition and the voluntary disclosure of greenhouse gas emission information. Table 4 shows the coefficient estimates for the testing of Hypothesis 1 resulting from regression with VDGHG as the dependent variable. As expected, the coefficient of the HHI is −0.0145 with a 1% significance level, which offers support for Hypothesis 1. 

#### 4.1.2. Discussion—Hypothesis 1

This significantly negative coefficient indicates that as industry competition increases, the cost is greater than the benefit of disclosure due to the increase in the proprietary cost related to disclosure, which can be interpreted as strategically less disclosure by managers seeking to maximize corporate value. Significant relations are also revealed between the voluntary disclosure of greenhouse gas emission information and the control variables. Some of the control variables (*SIZE*, *LEV*, *ROA*, *FO*, *MKT*) display a significant positive relationship with the voluntary disclosure level, and the others (*MTB*, *DA*, *MO*) show a significant negative association.

#### 4.1.3. Regression Results—Hypothesis 2

Through the empirical results of Hypothesis 1, it was confirmed that the higher the degree of competition in the industry, the greater the cost than the benefit from the disclosure, and the less voluntary disclosure was provided. Hypothesis 2 attempts to examine whether the relationship in Hypothesis 1 varies depending on corporate governance. Table 5 shows the results of analyzing whether the relationship between the degree of industry competition and the level of voluntary disclosure of greenhouse gas emission information differs according to corporate governance. As a result of the empirical analysis, it was found that the coefficient of the *HHI*, which represents the relationship between the degree of industry competition and the level of voluntary disclosure, had a statistically significant negative value at the 5% level (t-stat. = −2.49). Next, the coefficient of the interaction variable *HHI* × *CG*, which indicates the effect of corporate governance on the relationship between the degree of competition in the industry and the level of voluntary disclosure was found to have a significantly positive value at the 5% level (*HHI* × *CG*, t-stat. = 2.31), supporting Hypothesis 2. 

#### 4.1.4. Discussion—Hypothesis 2

This means that the better the corporate governance structure, the weaker the relationship between the degree of industry competition and the level of voluntary disclosure, and the higher the degree of industry competition, and the better the governance structure, the more voluntary disclosure. Summarizing the above empirical analysis results, it was found that the corporate governance structure weakened the negative relationship between the degree of industry competition and the level of voluntary disclosure of greenhouse gas emission information. These results can be interpreted that if competition is fierce in the industry, managers are more likely to choose a disclosure strategy to maximize corporate value over private benefits, and at this time, it can be interpreted that a good governance structure leads to such a manager’s decision to disclose because the benefits of voluntary disclosure on environmental issues are much greater than the proprietary cost of competition. In other words, it can be confirmed that a good governance structure plays a role in supporting managers to implement the optimal disclosure policy that can maximize the value of the company. Significant associations are also shown between VDGHG and the control variables. Some of the control variables (SIZE, LEV, FO, MKT) have a significant positive relationship with VDGHG, and others (MTB, ROA, DA, MO) show a negative association. 

### 4.2. The Effect of CG Components

In the main analysis of this study, empirical analysis was performed using the integrated measurement of corporate governance variables calculated based on shareholder rights protection, the board of directors, the disclosure of corporate governance, audit organizations, and management negligence allocation. Therefore, in additional analysis, the effect of the voluntary disclosure of greenhouse gas emission information was examined in consideration of the interaction with industry competition for each individual variable constituting the corporate governance structure. Through this, the validity of each governance variable used in this study is secured, and the distinguished effect on the voluntary disclosure of companies through interaction with competition for each governance variable is confirmed. 

Table 6 shows the analysis results by corporate governance component. As a result of the analysis, it was found that corporate governance interacted with industrial competition in the order of the disclosure of corporate governance (G3), shareholder rights protection (G1), and the board of directors (G2). In other words, the more active the disclosure of corporate governance, the stronger the protection of shareholder rights, and the more active the board of directors, the greater the impact on managers, inducing the voluntary disclosure of greenhouse gas emission information in industrial competition.

### 4.3. Robustness Regression

In this study, Hypothesis 1 and Hypothesis 2 were re-verified with a regression analysis model considering the fixed effect. This analysis is performed to reduce the effect of outlier bias in all analysis procedures. For example, the heterogeneity problem is somewhat eliminated by subtracting the group-level mean from each variable.

Table 7 shows the results of the robustness analysis. As a result of the empirical analysis, the coefficient for the HHI was −0.0145, which is a significant value at 5%, supporting Hypothesis 1. The coefficient for the interaction term of *HHI* × *CG* is positive (0.0350) at the 5% significance level, which is the result of supporting Hypothesis 2. Taken together, as can be seen in Table 6, the results of this paper show consistent results with major regression analysis even when analyzed through robustness tests.

## 5. Conclusions

This study identified the relationship between industry competition and the voluntary disclosure level of managers and examined how corporate governance affects these associations. Because the increase in industry competition to which the company belongs deteriorates its corporate profitability and increases the risk of bankruptcy and the possibility of managerial replacement, managers in highly competitive industries have a strong incentive to take strategic actions to increase profits and lower costs to improve their competitive status. This increase in strategic incentives for managers can appear as various types of competitive strategies, but among them, disclosure can directly affect the competitive position of a company. In addition, the voluntary disclosure policy of managers may vary depending on the competitive environment facing the company. Therefore, in this study, it was expected that the manager’s voluntary disclosure policy may vary depending on the degree of competition, and the relationship between industry competition and the corporate voluntary disclosure level was empirically verified. At this time, in order to measure the level of voluntary disclosure, whether to voluntarily disclose greenhouse gas emission information was used as a dependent variable.

Next, this study examined whether the relationship between the degree of industry competition and the level of voluntary disclosure differs according to the corporate governance structure. Studies examining the relationship between the existing corporate governance structure and the corporate disclosure level reported that a good governance structure increases the disclosure level by effectively monitoring and supervising managers to prevent them from trying to pursue private benefits and hiding them. According to this, regardless of the manager’s strategic intention according to the degree of competition, the higher the level of disclosure will be required as the company has a good governance structure. However, the disclosure of corporate information can negatively affect companies in industries where competition is fierce due to the existence of proprietary costs. In addition, in competitive industries, managers’ disclosure policies are likely to be an option to maximize corporate value rather than opportunistic actions to pursue their personal interests. Therefore, since the purpose of corporate governance is ultimately to maximize the value of the company, if the corporate governance structure is working properly, the governance structure will support managers to make optimal decisions. Based on these expectations, it was verified whether the relationship between the degree of competition in the industry and the manager’s disclosure policy varies according to the corporate governance structure.

The results of the empirical analysis were as follows. First, it was found that there was a negative relationship between the degree of competition in the industry and the level of voluntary disclosure of greenhouse gas emission information. These results mean that managers perform less voluntary disclosure to maximize the value of the company, as competition in the industry intensifies, the cost is greater than the benefit of disclosure due to the presence of proprietary costs. Second, it was found that the corporate governance structure weakens the relationship between the degree of industry competition and the level of voluntary disclosure. This can be interpreted that a good governance structure induces managers to have a disclosure strategy to maximize the value of the company rather than personal benefits. In other words, good corporate governance plays a role in helping companies implement these optimal disclosure policies because the benefits of voluntarily disclosing information about greenhouse gas emissions are much greater than the competition’s proprietary costs.

This study has differentiated contributions from previous studies. First, this study empirically analyzed the effect of industry-level competition on corporate voluntary disclosure policies through interactions with corporate governance. Through this, it was confirmed that the more competitive the industry, the better governance complements managers to implement an efficient disclosure policy, suggesting that the role of the corporate governance that operates depends on the competitive situation the company is in. These results are expected to provide implications when establishing policies for regulatory agencies that expect the role of strong governance. In addition, this study is expected to expand existing studies examining the relationship between competition or governance and voluntary disclosure in that it confirmed that managers’ decision-making may vary depending on the interaction between competition and corporate governance. Second, there was a problem in that it was difficult to generalize empirical results, as previous studies mainly used general fair disclosure as a measure of the voluntary disclosure level in verifying the relationship between the competition level and voluntary disclosure in the industry. On the contrary, this study is meaningful in that it provided robustness to the empirical results by measuring the more specific level of voluntary disclosure based on greenhouse gas emissions information. Finally, this study is expected to help capital market participants understand the incentives of managers to disclose voluntarily by reporting that industry competition is related to managers’ voluntary disclosure decisions.

In closing, the results of this study are subject to the following limitations. First, since we concentrate on limited companies in a single country, our evidence of an overall negative relationship between industry-level competition and the voluntary disclosure of greenhouse gas emissions information may not generalize to companies in other countries that are subject to more severe competition in industry than South Korea. Second, we are unable to identify the exact individual competition levels of companies because of measurement constraints for competition. Moreover, we used the integrated measurements for the corporate governance index in this study even if it was supplemented through additional analysis using component variables. All of the aforementioned facts may cause endogenous issues. 

Nonetheless, this paper is significantly noteworthy, as this is the first paper to investigate the effect of industry competition on the voluntary disclosure of greenhouse gas emissions information in South Korea. In future studies, it would be interesting to explore how much the volume of greenhouse gas emissions decreases with the level of industry competition or how specific dimensions of corporate governance affect this relationship.

## Figures and Tables

**Table 1:** The Data Selection Process.

Firm year observations from 2014 to 2020 with December closing fiscal year	14,536
Less:	
No data for control variables	2509
Missing data for corporate governance quality index	2621
Final observation	9406

**Table 2:** Descriptive Statistics.

Variables	Mean	STD	Q1	Median	Q3
*VDGHG*	0.0488	0.2155	0.0000	0.0000	0.0000
*HHI*	0.5538	0.4971	0.0000	1.0000	1.0000
*CG*	3.3288	0.3114	3.1780	3.3672	3.5263

Notes: Variable definition: *VDGHG* = 1 if a firm voluntarily discloses greenhouse gas emissions information; 0 otherwise; *HHI* = 1 if a firm is included in a competitive industry; 0 otherwise; *CG* = corporate governance index by KCGS.

**Table 3:** Pearson Correlation.

	(1)	(2)	(3)
(1) VDGHG	1.0000	0.0305	0.3298
		<0.0001	<0.0001
(2) HHI		1.0000	0.3431
			<0.0001
(3) CG			1.0000
		

**Table 4:** The regression result of Hypothesis 1.

Variables	Coeff.	t-stat.
*Intercept*	−1.7698	−31.78 ***
*HHI*	−0.0145	−2.97 ***
*SIZE*	0.0706	31.76 ***
*LEV*	0.0218	8.42 ***
*MTB*	−0.0191	−12.31 ***
*ROA*	0.0042	0.32
*DA*	−0.1485	−3.98 ***
*MO*	−0.1137	−7.60 ***
*FO*	0.1768	6.99 ***
*MKT*	0.0207	3.55 ***
*IND Dummy*	Included	
*YR Dummy*	Included	
F-value	154.99 ***	
Adj. R^2^	0.266	
Observations	6346	

(1) *** indicates significance at the 1% level. (2) *VDGHG* = 1 if a firm voluntarily discloses greenhouse gas emissions information; 0 otherwise; *HHI* = 1 if a firm is included in a competitive industry; 0 otherwise; *SIZE* = the natural logarithm of total assets; *LEV* = total debt/total asset; *MTB* = market value of equity/book value of equity; *ROA* = net income/total asset; *DA* = earnings quality measured by discretionary accruals; *MO* = majority shareholders’ ownership; *FO* = foreign investors’ ownership; *MKT* = 1 if a firm is included in the KOSPI market; 0 otherwise.

**Table 5:** The regression result of Hypothesis 2.

Variables	Coeff.	t-stat.
*Intercept*	−1.8110	−27.56 ***
*HHI*	−0.1301	−2.49 **
*CG*	0.0261	2.02 **
*HHI* × *CG*	0.0350	2.31 **
*SIZE*	0.0683	29.55 ***
*LEV*	0.0209	7.96 ***
*MTB*	−0.0182	−11.52 ***
*ROA*	0.0026	−0.20
*DA*	−0.1521	−4.07 ***
*MO*	−0.1138	−7.56 ***
*FO*	0.1601	6.31 ***
*MKT*	0.0182	3.07 ***
*IND Dummy*	Included	
*YR Dummy*	Included	
F-value	92.82 ***	
Adj. R^2^	0.273	
Observations	6346	

(1) ** and *** indicate significance at the 5%, and 1% levels, respectively. (2) *CG* = corporate governance index by KCGS. (3) See Table 4 for definitions of other variables.

**Table 6:** The effect of ESG investments.

Variables	Coeff.	t-stat.
*Intercept*	−1.7768	−16.95 ***
*HHI*	−0.0664	−0.76
*G1*	−0.0052	−0.20
*G2*	0.0076	0.46
*G3*	0.0005	0.03
*G4*	0.0291	1.43
*G5*	0.0016	0.10
*HHI × G1*	0.0615	3.20 ***
*HHI × G2*	0.0341	2.97 ***
*HHI × G3*	0.0492	3.66 ***
*HHI × G4*	−0.0143	−0.99
*HHI × G5*	−0.0004	−0.04
*Control Variables*	Included	
F-value	54.92 ***	
Adj. R^2^	0.307	
observations	3409	

(1) *** indicates significance at the 1% level. (2) *G1* = shareholder rights protection; *G2* = board of directors; *G3* = disclosures of corporate governance; *G4* = audit organizations; *G5* = management negligence allocation. (3) See Table 4 for definitions of other variables.

**Table 7:** Robustness Regression.

Panel A. H1		
**Variables**	**Coeff.**	**t-stat.**
*HHI*	−0.0145	−2.97 ***
*Control Variables*	Included	
F-value	173.08 ***	
Adj. R^2^	0.304	
Observations	6346	
**Panel B. H2**		
**Variables**	**Coeff.**	**t-stat.**
*HHI*	−0.1301	−2.49 **
*CG*	0.0261	2.02 **
*HHI × CG*	0.0350	2.31 **
*Control Variables*	Included	
F-value	106.00 ***	
Adj. R^2^	0.311	
Observations	6346	

(1) **, and *** indicate significance at the 5%, and 1% levels, respectively. (2) See Table 4 and Table 5 for definitions of other variables.

## Data Availability

Not applicable.
